# Effects of Cinnamon Essential Oil on Intestinal Flora Regulation of Ulcerative Colitis Mice Colonized by *Candida albicans*

**DOI:** 10.3390/microorganisms13122724

**Published:** 2025-11-28

**Authors:** Yuan Gao, Silin Liu, Jinhe Wang, Yan Xu, Yuyang Guo, Zesen Fang, Fuling Wang, Jianghan Luo, Lijun Yan

**Affiliations:** School of Pharmacy, Engineering Research Center of Natural Antineoplastic Drugs, Ministry of Education, Harbin University of Commerce, Harbin 150076, China; ssrp1018@163.com (S.L.); wangjinhe2025@163.com (J.W.); 18252583359@163.com (Y.X.); 18846086109@163.com (Y.G.); 15610144007@163.com (Z.F.); iwfl86@163.com (F.W.); 102690@hrb.edu.cn (J.L.); ylj@hrbcu.edu.cn (L.Y.)

**Keywords:** cinnamon essential oil, *Candida albicans*, ulcerative colitis, intestinal flora, JAK-STAT pathway

## Abstract

Cinnamon essential oil (CEO), a safe, medicinal, and edible Traditional Chinese Medicine (TCM) component, was investigated for its anti-*Candida albicans* property and ability to relieve intestinal inflammation. The anti-*Candida albicans* ability of CEO was evaluated by Minimum Inhibitory Concentration (MIC), 2,3-Bis-(2-Methoxy-4-nitro-5-sulfophenyl)-2H-tetrazolium-5-carboxanilide (XTT), and Scanning Electron Microscope (SEM) methods. By constructing ulcerative colitis (UC) mice intestinally colonized by *C. albicans*, the CEO effects on the regulation of flora, the relief of intestinal inflammation, and possible related signal pathway were discussed. The results showed that CEO has a significant effect on inhibiting *C. albicans*, where the MIC_80_ value was 265 μg/mL, and the Sessile Minimum Inhibitory Concentration (SMIC)_80_ value was 530 μg/mL. SEM showed the CEO could inhibit *C. albicans* mycelium growth and biofilm formation. CEO can regulate the flora disturbance, reduce inflammatory factors level, and play a protective role in intestinal mucosal damage. Network pharmacology predicts CEO may be associated with Janus Kinase-Signal Transducer and Activator of Transcription (JAK-STAT) pathway. It was proved that CEO had an inhibitory effect on the JAK-STAT pathway by qPCR determination. These findings suggest CEO may have therapeutic potential for *C. albicans*–associated UC.

## 1. Introduction

Ulcerative colitis (UC) is a chronic nonspecific inflammatory bowel disease that primarily affects the colon and rectum. It is characterized by a prolonged disease course, difficulty in achieving complete remission, and a high rate of recurrence [1]. The exact etiology of UC remains unclear, although it is widely considered to involve a complex interplay of genetic predisposition, environmental triggers, gut microbiota dysbiosis, and immune system dysfunction [2]. Emerging evidence highlights the critical role of intestinal microbiota, including both bacterial and fungal communities, and their associated metabolic pathways and molecular mechanisms in modulating the intestinal mucosal barrier integrity and innate immune responses in UC [3].

The gut microbiota are a complex ecosystem dominated by bacteria, which antagonize or cooperate with fungal flora such as *C. albicans* to maintain intestinal homeostasis [4]. *C. albicans* can colonize diverse mucosal surfaces such as the oral cavity and vagina; nevertheless, most evidence indicates that the gastrointestinal tract is the main portal through which *C. albicans* enters the bloodstream [5,6,7]. *C. albicans* helps to shape a healthy intestinal homeostasis environment by inhibiting multiple dominant bacteria genera and local inflammation in the gut [8,9]. However, its overgrowth can also promote inflammation, induce diseases, and delay wound healing [10]. *C. albicans* interacts with the mucosa through adhesion, invasion, tissue damage, and apoptosis [11], and its overgrowth is tightly linked to inflammatory bowel disease (IBD) [12,13]. Dysbiosis weakens the mucosal barrier, triggers inflammatory cytokine release, and accelerates UC.

Cinnamon essential oil (CEO) is the volatile oil extracted from the dried branches and leaves of cinnamon, a kind of Chinese herbal medicine with medicinal and edible functions. CEO is a volatile, hydrophobic, mild, and viscous liquid that is usually yellowish with a sweet spicy odor [14]. At present, about 127 components from CEO have been isolated and identified [15,16]. The main component is (E)-cinnamaldehyde, which accounts for 70–90% [17,18]. CEO has anti-inflammatory, antibacterial, antioxidant, antitumor, and other pharmacological activities [19,20,21]. CEO has an inhibitory effect on *C. albicans* in vitro, and it may also rebalance the gut microbiota by increasing probiotics like *Bifidobacterium* while suppressing *Escherichia coli* and *Enterococcus* and dampen inflammation via downregulation of IL-1β and TNF-α. Additionally, the IL-6–mediated JAK-STAT pathway, through STAT3 phosphorylation and RORγt synthesis, modulates the Th17/Treg axis and contributes to anti-*Candida* immunity [22]. Despite these insights, the effects of CEO in inhibiting *C. albicans* in mice and the UC effects on *C. albicans* colonization need to be further studied. The mechanism of CEO on both inflammatory injury and microbial ecology in *C. albicans*–colonized UC remains poorly understood. In this study, we established a UC mouse model under *C. albicans* colonization to systematically evaluate the therapeutic potential of CEO.

## 2. Materials and Methods

### 2.1. Strain and Chemicals

*C. albicans* (strain number 250, SN250) was generously provided by Professor Chen Changbin. SPF BALB/c mice, 20 ± 2 g, male, were purchased from Biotechnology Co., Ltd., (Benxi, China), license No. SCXK (Liao)-2020-0001. This experiment received approval from the Experimental Animal Ethics Committee of Harbin University of Commerce (HSDYXY-2022014). The CEO was acquired from the China National Institute for Food and Drug Control with 95% content, lot number: 111550-200001, CAS: 8000-80-5, Beijing, China. Mesalazine was purchased from Sunflower Pharmaceutical Co., Ltd., (Harbin, China).

### 2.2. Effect of Cinnamon Essential Oil on Anti-Candida albicans Biofilm

In accordance with the micro-dilution method outlined in the M27-A protocol established by the National Committee for Clinical Laboratory Standards (NCCLS), now known as the Clinical and Laboratory Standards Institute (CLSI), the *C. albicans* suspension was prepared at a concentration of 2 × 10^3^ CFU/mL. Different concentrations of CEO were dispensed in 100 μL aliquots into individual wells of a 96-well plate, with saline serving as the negative control [23]. The blank wells contained yeast extract peptone dextrose (YPD) medium alone, while the other wells received 100 μL of the fungal suspension. Following incubation at 30 °C for 48 h, the optical density at 630 nm was measured to determine the MIC_80_, defined as the lowest CEO concentration that inhibited 80% of *C. albicans* growth.

To establish a biofilm of *C. albicans*, the fungal suspension was adjusted to a concentration of 2 × 10^6^ CFU/mL using RPMI 1640 medium. Various concentrations of CEO were added to a 100 μL fungal suspension in a 96-well plate. The plate was cultured at 37 °C for 24 h, gently rinsed with PBS buffer three times, and then treated with 200 µL of XTT-menadione solution. Following incubation in the dark at 37 °C for 2 h, the supernatant was measured at OD_490 nm_. The SMIC_80_ was determined, denoting the drug concentration in the lowest well with an 80% decrease in OD value compared to the negative control [24,25].

Biofilm inhibition rate (%) = (1 − OD treatment group/OD negative group) × 100%

### 2.3. Effect of Cinnamon Essential Oil on Mycelial Morphology of Candida albicans

*C. albicans* suspensions (2 × 10^6^ CFU/mL) were treated with CEO at concentrations corresponding to 2 × SMIC_80_, SMIC_80_, and 0.5 × SMIC_80_ and incubated at 37 °C for 12 h. After the suspensions were centrifuged at 3000 rpm for 3 min, the resulting pellets were resuspended in 600 μL of 2.5% glutaraldehyde and fixed overnight at 4 °C in the dark. After the glutaraldehyde solution was discarded, *C. albicans* were washed 3 times with PBS; afterwards, the samples were subjected to graded ethanol dehydration (30%, 50%, 70%, 90%, and 100%), with each concentration applied for 15 min, and finally dried overnight in a vacuum desiccator. The *C. albicans* samples were then vacuumed, gilded, and observed using SEM, JEOL (Tokyo, Japan) [26,27].

### 2.4. Establishment of Ulcerative Colitis Mice Model Colonized by Candida albicans

The ulcerative colitis (UC) mice model colonized by *C. albicans* was established by the administration of *C. albicans* suspension and free drinking of 3% Dextran Sulfate Sodium (DSS) solution [28,29]. BALB/c mice were fed at a constant temperature and humidity for 1 week. Distilled water and food were given regularly, with daily monitoring of the mice’s health status. A total of 70 mice were randomly divided into 7 groups: a control group, a DSS group, a model group (DSS+ *Candida albicans* (C.A) group), 3 drug administration groups (low-dose, medium-dose and high-dose CEO groups), and a positive drug (mesalazine) group. Each group consisted of 10 animals.

The sample size of 10 mice per group was determined through statistical power analysis to ensure robust statistical power while complying with the 3R principles. An a priori power analysis was conducted using G*Power 3.1.9.7. With the parameters set as effect size d = 1.2, significance level α = 0.05, statistical power (1 − β) = 0.80, and number of groups = 7, the ANOVA fixed-effects model calculation yielded a minimum sample size of 8 animals per group. Accounting for a potential 20% attrition rate, the final sample size was set at 10 mice per group, totaling 70 mice, thereby ensuring the minimal use of animals while fulfilling the statistical requirements.

Mice in the control group drank distilled water freely without any additional treatment. In the DSS group, mice drank distilled water freely on days 1–4, and 3% DSS solution on days 5–11. Mice in the model group also drank distilled water freely but received intragastric administration of C.A (10^8^ CFU/animal/day) on days 1–4 and 3% DSS solution on days 5–11. The mice in both the administration and the positive groups had free access to distilled water; however, they received an intragastric dose of C.A (10^8^ CFU/animal/day) on days 1–4 and transitioned to consumption of a 3% DSS solution freely on days 5–11. During days 5–11, the mice in the administration group were additionally gavaged with CEO at doses of either 5.3 mg/kg/d (low), 10.5 mg/kg/d (medium), or 21 mg/kg/d (high). Meanwhile, the positive group was treated with mesalazine at a dosage of 200 mg/kg/d. Both the DSS group and model group (DSS+C.A) received an equal volume of normal saline. All groups of the mice were sacrificed on day 12 for sample collection.

### 2.5. The Fecal Candida albicans Load in Mice Treated with Cinnamon Essential Oil

The feces of mice in each group were collected at days 1, 3, 5, 7, 9 and 11, dried and weighed, and then resuspended in pre-cooled PBS (pH 7.4) and coated on Chromogenic medium containing chloramphenicol. After incubation at 37 °C for 48 h, the number of *C. albicans* microcolonies on the plate was quantified (CFU/mg).

### 2.6. Colonic Edema, Weight to Length Ratio, and Immune Organ Index

All mice were weighed daily, and observations regarding the fecal characteristics, fur condition, activity levels, and mortality were systematically recorded. The colon, spleen, and thymus of the euthanized mice were collected after 12 days. The colon length and weight were measured, and the colonic edema index and colonic weight–length ratio were calculated. The immune organ index was calculated to evaluate the immune system status of the mice.

Colon edema index = colon weight (g)/body weight (g);

Colon weight to length ratio = colon weight (g)/colon length (cm);

Immune organ index = Immune organ (spleen, thymus) weight (g)/body weight (g)

### 2.7. Hematoxylin–Eosin and Periodic Acid–Schiff Staining

The colon tissues were placed in embedding cassettes and fixed in 10% formalin solution at 4 °C for one week prior to the subsequent staining experiments. Following fixation, the tissues underwent ethanol gradient dehydration, paraffin embedding, sectioning, and staining with hematoxylin–eosin (HE) and periodic acid–Schiff (PAS) methods. Finally, the stained sections were mounted with resinous medium, then the appropriate ratio was adjusted under the microscope and photographed.

### 2.8. IL-6, IL-8, and IL-17a Determination

The concentrations of interleukin-6 (IL-6), interleukin-8 (IL-8), and interleukin-17A (IL-17a) were quantified using a double-antibody sandwich ELISA. The microplates were coated with purified antibodies, followed by the addition of standards for IL-6, IL-8, and IL-17A. Subsequently, corresponding antibodies labeled with horseradish peroxidase (HRP) were applied. After incubation and washing steps, the enzymatic reaction was initiated by adding the 3,3′,5,5′-Tetramethylbenzidine (TMB) substrate to facilitate chromogenic development. The TMB is catalyzed by HRP enzymes, resulting in a blue color, and is converted to the final yellow color by interaction with the acid color development stop solution. The coloration observed in the microplate was correlated with the levels of IL-6, IL-8, and IL-17a. The absorbance value at 450 nm was measured using a microplate reader, and the concentrations of IL-6, IL-8, and IL-17a in the supernatants of the colon tissue were determined based on a standard curve.

### 2.9. Sequencing of Intestinal Flora of Mice Colonized by Candida albicans

Genomic DNA from the fecal microbial community was isolated and subjected to polymerase chain reaction (PCR) amplification with primers 338F (5′-ACTCCTACGGGAGGCAGCAG-3′) and 806R (5′-GGACTACHVGGGTWTCTAAT-3′). The amplification aimed to detect the 16S rRNA gene within the V3-V4 hypervariable region. The resulting amplicons were sequenced on an Illumina MiSeq platform (Illumina, San Diego, CA, USA), following the standard protocols provided by Shanghai Majorbio Bio-pharm Technology Co., Ltd., Shanghai, China.

### 2.10. Target and Mechanism Prediction of Cinnamon Essential Oil Against Candida albicans Colonization in Ulcerative Colitis

The published database was searched to screen the components of CEO, combined with the TCMSP [30] database with OB ≥ 30% and DL ≥ 0.18 to obtain the active components of CEO. The target of the relevant chemical composition was then obtained using the TCMSP and PharmMapper [31] databases, and the “Mouse” gene bank was downloaded using the Uniprot database [32]. Microsoft Excel version 2308 was used for target protein normalization and protein–gene conversion.

Cytoscape 3.8.2 [33] software was utilized to construct a network diagram illustrating the active components of CEO and their corresponding action targets. Employing “Ulcerative colitis associated with *Candida albicans*” as disease-related keywords, Genecards and Drugbank disease databases were used to screen disease-related genes and proteins and obtain related targets.

The disease targets were imported into the STRING 11.5 database [34] to construct the protein–protein interaction (PPI) network. GO biological function analysis and KEGG pathway enrichment analysis were conducted using the DAVID database [35,36]. The pathway network diagram of CEO and *C. albicans* colonization in UC was constructed by Cytoscape 3.8.2.

### 2.11. Quantitative Reverse Transcription–Polymerase Chain Reaction Assay

Total RNA was isolated using Trizol, Ambion, (Austin, TX, USA). Quantitative real-time PCR (qRT-PCR) was performed in duplicate on an Applied Biosystems 7500 Fast Real-Time PCR system, Thermofisher, (Waltham, MA, USA) utilizing SYBR Green qPCR Mix (Beyotime, Shanghai China). All data were analyzed with QuantStudio™ Design & Analysis Software Version 1.6. The primers employed in the PCR are listed in Table 1. The PCR reaction cycles were as follows: an initial denaturation at 95 °C for 10 min, followed by 40 cycles of denaturation at 95 °C for 15 s, annealing at 60 °C for 30 s, and extension at 72 °C for 30 s. The ΔCT values of negative control group were obtained, and the relative gene expression multiple was expressed by the 2^−ΔΔCT^ method.

## 3. Results

### 3.1. Effect of Cinnamon Essential Oil on the Formation of Candida albicans Biofilm

**Figure 1:**
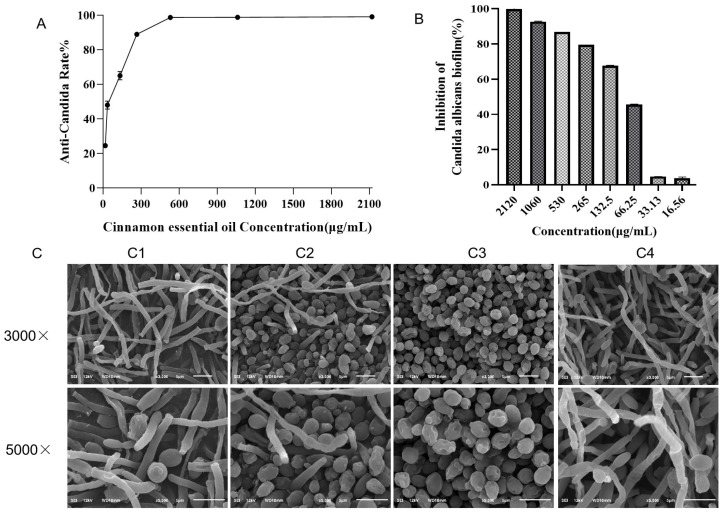
Anti-*Candida* rate% of CEO (**A**); anti-*Candida* biofilm rate of CEO (%) (**B**); morphology of *C*. *albicans* at 12 h by SEM (**C**). C1: 265 μg/mL (CEO); C2: 530 μg/mL (CEO); C3: 1060 μg/mL (CEO); C4: negative control.

With the increasing concentration of CEO, the inhibitory effect on *C. albicans* is also enhanced. Figure 1A showed that the MIC_80_ was 260 μg/mL. When the CEO concentration reached 530 μg/mL, the preformed biofilms showed an 80% reduction in absorbance (SMIC_80_ = 530 μg/mL) (Figure 1A,B).

SEM results showed dense intertwined hyphae in the control group and a typical signal before the mature biofilms, whereas CEO treatment disrupted the hyphal formation and surface structure. In the 265 μg/mL group, there was a certain amount of mycelia formation in *C. albicans*, but the mycelia length was short, and yeast cells could be observed. In the 530 μg/mL, there were very few mycelia formation, most of *C. albicans* existed in the form of yeast cells, and some of the yeast cells had pits on the surface. *C. albicans* in the 1060 μg/mL group had no mycelia formation, all existed in the form of yeast cells, and the surface of yeast cells showed serious depression and folds (Figure 1C).

### 3.2. Effects of Cinnamon Essential Oil on Status and Fecal Candida albicans Load in Ulcerative Colitis Mice

The control group of mice had glossy hair, a normal diet, good mental state, no bloody stool, and no colon edema. On day 8, the model group and the low-, medium-, and high-dose CEO group showed slow movement, reduced food intake, and soft and unformed feces, but compared to the model group, the mice treated with CEO exhibited a notable improvement. The therapeutic effect was more pronounced in the high-dose CEO group. With the consumption of the DSS solution, the mice gradually developed bloody stools. The mice status was relieved with CEO, and the high-dose CEO group had the most obvious therapeutic effect. The physical signs of the DSS group were similar to the model group, but bloody stool appeared slightly. No mice died during administration.

**Figure 2:**
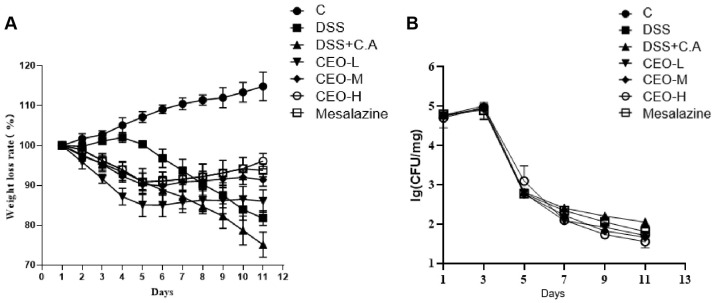
Ratio of weight loss in mice (**A**); effect of CEO on the amount of C.A in the intestinal contents of mice (**B**). Note: C is the blank group; DSS is the DSS group; DSS+C.A is the model group; CEO-L is the low-dose group of CEO; CEO-M is the medium-dose group of CEO; CEO-H is the high-dose group of CEO.

The body weight of the model group decreased, while that of the control group increased. In the first 4 days, the weight of the low-, medium-, and high-dose CEO and mesalazine groups showed a decreasing trend, which proved that the *C. albicans* had a certain effect. The decline slowed on day 4 and increased after day 7, demonstrating that CEO had some relief on weight loss in mice caused by *C. albicans* (Figure 2A).

*C. albicans* showed a blue smooth colony after culture in Candida Chromogenic medium. *C. albicans* colonies were observed after culture. On day 1 and 3, the number of C. *albicans* showed an increasing trend, and from day 4, after drinking DSS solution freely and being treated with CEO, the number of *C. albicans* in the high-dose group showed a significant decreasing trend (Figure 2B).

### 3.3. Effects of Cinnamon Essential Oil on Colon Tissue and Inflammatory Factors

**Figure 3:**
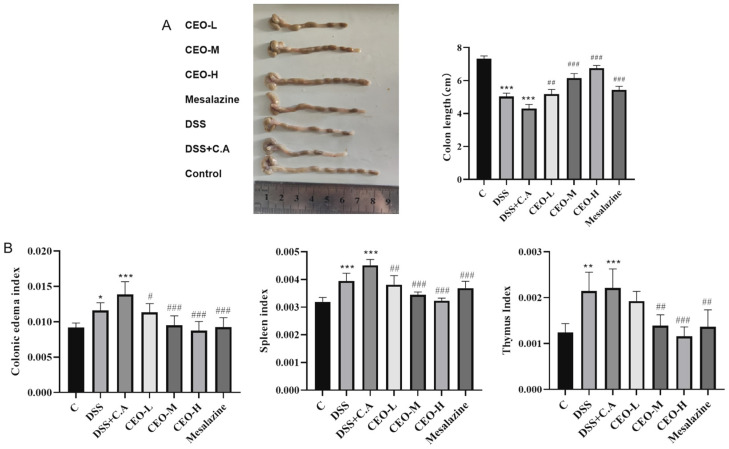
Mice colon length (**A**); colonic edema index, spleen index and thymus index in mice (**B**). Note: compared with control group (* *p* < 0.05, ** *p* < 0.01, and *** *p* < 0.001) and compared with model group (# *p* < 0.05, ## *p* < 0.01, and ### *p* < 0.001).

Compared to the control group, the colons of both the DSS and model groups exhibited a significant reduction in length. In comparison to the model group, the mice in the CEO treatment group showed an alleviation of symptoms, with the high-dose CEO group demonstrating the most pronounced effects. This indicates that CEO has potential therapeutic benefits in mitigating the colon shortening associated with UC (Figure 3A).

Compared to the control group, both the colonic edema index and immune organ index in the DSS and model groups of mice were significantly elevated. This finding confirms that UC induces colonic edema and severe inflammation in these mice. In contrast, when compared to the model group, there was a significant reduction in both the colonic edema index and immune organ index within the CEO treatment group, particularly noted in the high-dose group. These results indicate that CEO exhibits a notable therapeutic effect on colonic edema and the enlargement of immune organs associated with UC colonization by *C. albicans* (Figure 3B).

**Figure 4:**
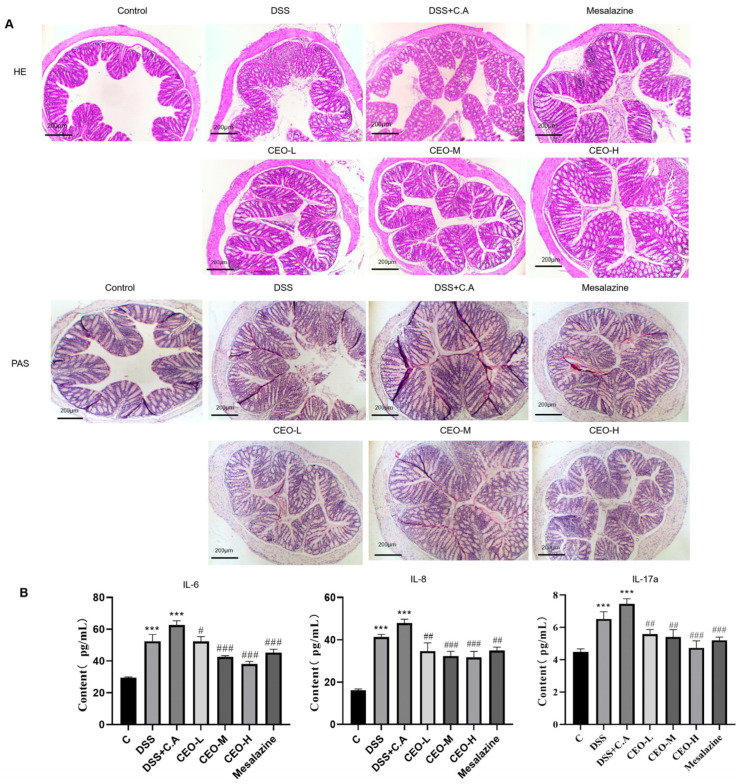
HE staining and PAS staining of mice colons 40× (**A**); effect of CEO on the content of IL-6, IL-8, and IL-17a in mouse colon tissue (**B**). Note: compared with control group (*** *p* < 0.001) and compared with model group (# *p* < 0.05, ## *p* < 0.01, and ### *p* < 0.001).

The control group revealed the colon mucosa structure was normal, the large intestine glands were tightly spaced, the epithelial cells had no ulcers, and there were more goblet cells. The DSS group and the model group showed the acute inflammatory reaction of mucosal erosion, congestion, edema, and reduced crypts and neutrophils and other inflammatory cell infiltration, which were more obvious in the model group. The colonic mucosal structure improved in the mesalazine group, and there was no visible ulcer, epithelial cell destruction, or inflammatory cell infiltration. The colonic mucosal structure was improved, and no obvious ulcers were found in the high-dose group. There was no visible ulcer or epithelial cell injury, the goblet cells were decreased, and the intestinal mucosal structure was also improved in the medium dosage CEO group. Even though there were no visible ulcers and the colonic mucosal structure improved in the low dosage CEO group, goblet cells and local glandular epithelial cells still suffered damage (Figure 4A).

Compared to the control group, a significant number of purplish-red *C. albicans* spores and mycelia were observed in the colonic mucosa and submucosa of mice in both the DSS and model groups, with more pronounced findings noted in the model group than in the DSS group. After treatment with CEO, the quantities of *C. albicans* spores and mycelia exhibited varying degrees of reduction compared to the model group. Notably, the medium- and high-dose groups of both CEO and mesalazine demonstrated the most pronounced effects (Figure 4A).

Compared to the model group, IL-6 content in the high-dose and medium-dose groups decreased significantly; the IL-8 content in the CEO treatment group was reduced, the reduction degree was proportional to the dose concentration, and the IL-17a content was decreased in the high-dose and mesalazine groups and also decreased in the medium-dose and low-dose CEO groups (Figure 4B). It was speculated that in colon tissue of mice, the CEO could reduce the IL-6, IL-8, and IL-17a content.

### 3.4. Effects of Cinnamon Essential Oil on Intestinal Flora of Ulcerative Colitis Mice

**Figure 5:**
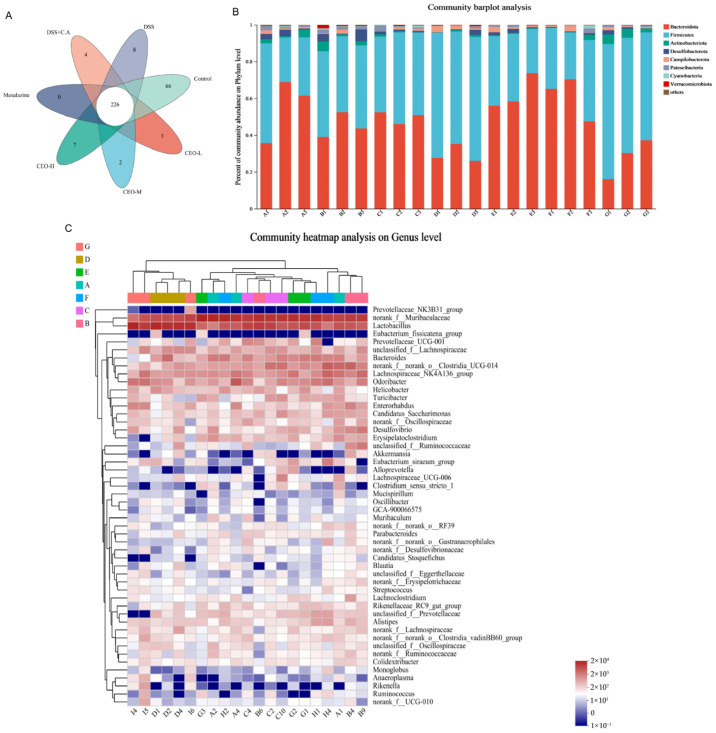
Venn diagram of the species composition of each group of intestinal flora (**A**); histogram of abundance on the phylum level of the intestinal flora (**B**); heat map analysis of species clustering at the genus level of the intestinal flora of each sample (**C**). Note: A: CEO low-dose group; B: CEO medium-dose group; C: CEO high-dose group; D: mesalazine group; E: DSS+C.A group; F: DSS group; G: control group.

Principal component analysis (PCA) was employed to assess the variations in bacterial abundance across the samples. The total number of specific OTUs within each group is as follows: CEO-L group 3/403; CEO-M Group 2/405; CEO-H group 7/417; mesalazine group 0/356; model group 4/400; DSS group 8/400; control group 66/493. Compared to the control group, the species richness of intestinal flora in the model group exhibited a decrease. In contrast, when compared to the model group, an improvement in species richness was observed in both the CEO group and the mesalazine group (Figure 5A).

The results of the species analysis indicated that a total of eight phyla were identified at the phylum classification level, with Bacteroidota and Firmicutes comprising the largest proportions. Compared to the control group, the model group exhibited an increased abundance of Bacteroidota, Campylobacter, and Verrucomicrobia. Conversely, there was a decrease in the abundance of Firmicutes, Actinobacteriota, Escherichia coli, and Cyanobacteria within the model group. Compared to the model group, the abundance of Bacteroidota, Desulfuricutes, Campylobacter, Escherichia coli, and Verrucomicrobia was found to be elevated in both the CEO group and the mesalazine group. Notably, there was a significant increase in the abundance of Verrucomicrobia. The abundances of Firmicutes, Actinobacteriota, and Cyanobacteria were decreased, and the abundances of Actinobacteriota in the CEO-M group and mesalazine group were significantly decreased. The relative abundance analysis results of intestinal flora classification at the phylum level are shown in Figure 5B.

**Figure 6:**
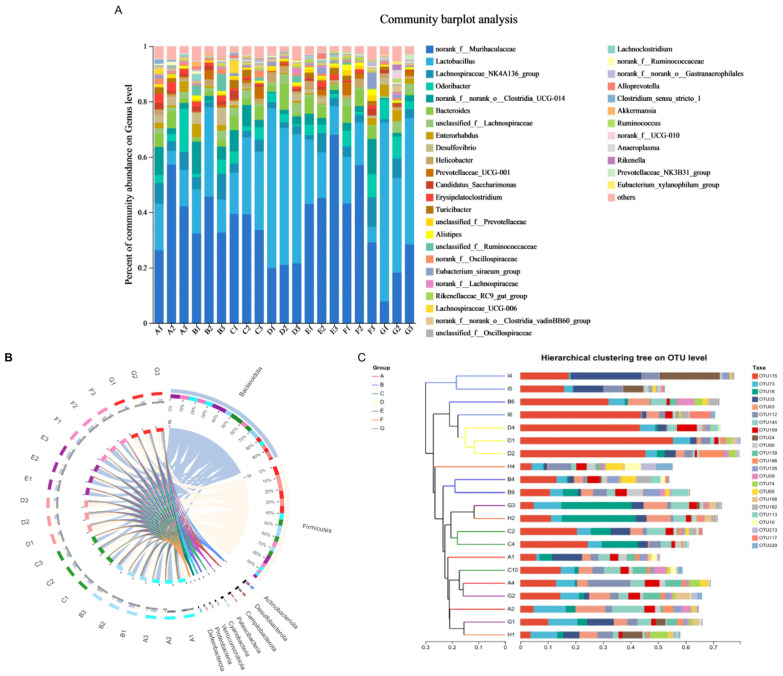
Histogram of abundance at the genus level of intestinal flora (**A**); analysis of the Circos of each group of intestinal flora species at the genus level (**B**); intestinal flora cluster analysis (**C**). Note: A: CEO low-dose group; B: CEO medium-dose group; C: CEO high-dose group; D: mesalazine group; E: DSS+C.A group; F: DSS group; G: control group.

The results of the multilevel cluster analysis and species analysis at the genus level are shown in Figure 5C and Figure 6A. The dominant genus in the control group was *Lactobacillus*, which accounted for the largest abundance, followed by norank_f__Muribuculacease. Compared with the control group, the bacterial flora in the model group changed significantly, and the number of bacteria norank_f__ Muribuculacease and *Bacteroides* was mainly significantly increased. The number of bacteria *Lactobacillus*, *Odoribacter*, and *Enterorhabdus* was mainly significantly reduced. Compared with the control group, the bacterial flora of the CEO group changed significantly, and the number of bacteria *Bacteroides* and *Enterorhabdus* was mainly significantly reduced. In addition, compared with the model group, the CEO group and mesalazine group changed significantly, the bacterial abundance of *Bacteroides*, *Lactobacillus*, lachnospiraceae_NK136A11_group, and *Robuteria* were significantly reduced, and the bacterial abundance of norank_f__norank_o__Clostridia_UCG-014 was significantly increased.

By using the Circos map, we can more intuitively understand the abundance size of genes or species in each sample, leading to a better understanding of the biological data. Compared to the control group, the model group exhibited a significant decrease in the proportions of Firmicutes and Actinobacteriota. Conversely, there was a notable increase in the proportions of Bacteroidota, Desulfobactorta, Verrucomicrobiota, and Deferribacterota. This indicates that the composition of the gut microbiota in the mice underwent alterations following the establishment of the model. In comparison to the model group, there was a significant increase in the proportion of Firmicutes-dominant bacteria within both the CEO group and mesalazine group. The results of the Circos analysis for each sample and species at the genus level are presented in Figure 6B.

The results of the clustering analysis (Figure 6C) indicated significant differences in species abundance distribution between the groups, as well as notable variations in species abundance distribution within each group. The difference between the groups was higher than that observed within each group. The model and control groups were clustered in distinct branches, with sample points exhibiting considerable separation from one another. This indicates significant differences in the community composition between the two groups. The mesalazine and CEO groups were positioned between the control group and the model group, suggesting that they may play a role in regulating and promoting the enhancement of intestinal flora in mice. The CEO-M group had a better effect than other CEO groups. In conclusion, CEO treatment reshapes the intestinal microbiota of DSS-induced UC mice colonized by *C. albicans*, markedly increasing the beneficial taxa while reducing harmful ones, thereby enhancing both the diversity and richness and conferring therapeutic benefit.

### 3.5. Target and Signal Pathway Prediction of Cinnamon Essential Oil on Ulcerative Colitis Mice

A total of 93 CEO components were obtained from the literature, and 18 active components were obtained after the literature supplement and OB and DL screening. After searching the disease database and supplementing the literature, a total of 490 disease targets associated with *C. albicans* colonization of UC were obtained after the translation, de-duplication, and deletion of duplicate targets.

**Figure 7:**
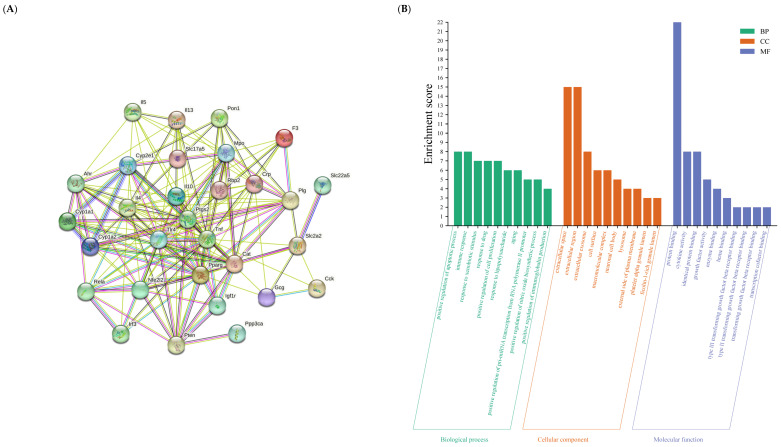

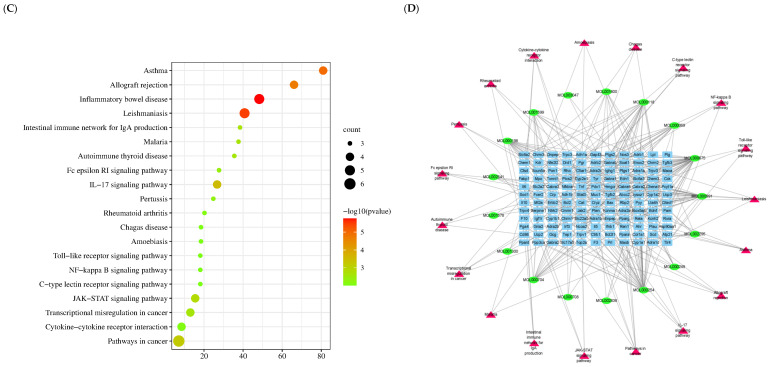
“Active ingredient of CEO-UC associated with *Candida albicans*” key target PPI network (**A**); GO enrichment analysis of the target of CEO in the treatment of UC (**B**); enrichment analysis on pathways of CEO in the treatment of UC (**C**); CEO-active ingredient-*Candida albicans* colonization UC pathway (**D**).

The PPI network maps of active components of CEO and *C. albicans* in UC were constructed by String. The results showed that CEO could directly or indirectly interact with 124 targets and 824 kinds of interactions. *C. albicans* colonizing UC can have direct or indirect interaction with 490 targets, and the interaction can reach 9204 kinds. We obtained 31 common targets of “active ingredient of CEO-*C. albicans* colonizing ulcerative colitis”. The key target protein PPI interaction of “Active Ingredient of CEO-*C. albicans* colonizing ulcerative colitis” is shown in Figure 7A. The network comprises 30 nodes and 158 edges.

The David database was utilized to analyze the signaling pathways associated with the relevant targets of CEO in the treatment of *C. albicans*-colonized ulcerative colitis (UC). The visual outcomes of the GO analysis included biological processes (BP), cellular components (CC), and molecular functions (MF). As illustrated in Figure 7B, the length of each horizontal bar chart corresponds to its gene count, while the color gradient ranging from orange to red signifies the Log10 (*p*) value, with smaller values represented by orange and larger values by red. The top ten entries within each classification have been selected for display. There are 185 BP-related items associated with various biological processes, including inflammatory response, immune response, positive regulation of smooth muscle cell proliferation, enhancement of immunoglobulin production, and the positive regulation of STAT protein tyrosine phosphorylation.

Additionally, these items are involved in the positive modulation of the JAK-STAT signaling cascade. There are 14 CC related items, involving phagocytic, receptor complex, synapse, etc. There are 34 items related to MF, involving DNA binding, cytokine activity, transcription coactivator binding, and so on. The results of the KEGG analysis visualization are presented in Figure 7C. The color gradient of the bubbles, ranging from green to red, indicates the Log10 (*p*) values, with smaller values represented by green and larger values by red. The size of each bubble reflects to the number of genes linked to the particular pathway, while the horizontal axis illustrates the proportion of genes within this pathway relative to the total number of genes analyzed. Signaling pathways include the JAK-STAT, IL-17, T cell receptor, C-type lectin receptor, Toll-like receptor, Th17 cell differentiation, and 69 other pathways. The active ingredient of CEO—*C. albicans* colonization of UC—is shown in Figure 7D.

### 3.6. Cinnamon Essential Oil Effects on JAK2-STAT3 Signaling Pathway

**Figure 8:**
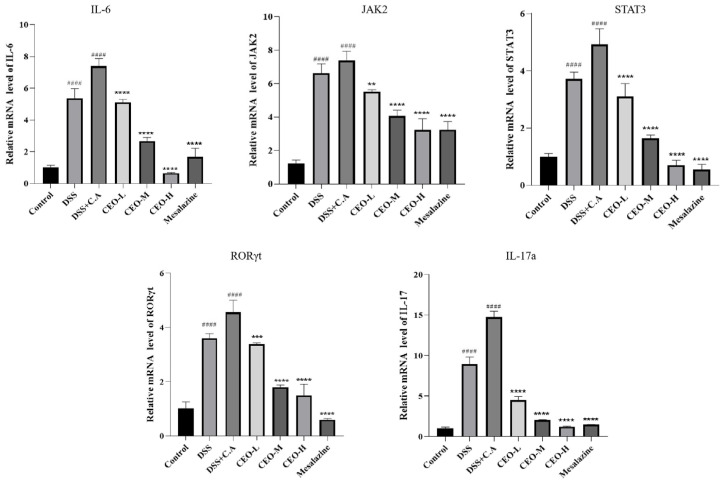
Gene expression of IL-6, JAK2, STAT3, RORγt, and IL-17 in colonic tissues. Note: compared with control group (#### *p* < 0.0001) and compared with model group (** *p* < 0.01, *** *p* < 0.001, **** *p* < 0.0001).

Activation of the JAK2-STAT3 signaling pathway is achieved through the expression of IL-6, which is involved in various physiological and pathological processes. qRT-PCR was employed to assess the effects of CEO on the expression levels of IL-6, JAK2, STAT3, RORγt, and IL-17A (Figure 8). The results indicated that, compared to the control group, there was an increase in the relative expression levels in both the DSS group and model group. Furthermore, when comparing the model group with the CEO treatment groups, a significant decrease in IL-6, JAK2, STAT3, RORγt, and IL-17A was observed. The IL-6 decreasing trend was correlated with the CEO concentration. The CEO-L group decreased by 91%. JAK2 expression decreased in CEO-treatment groups compared to the model group, and the CEO-H and mesalazine groups had similar effects. In the CEO-M group, the STAT3 decline was 67%, and in the CEO-H group, the decline was 86%; In the CEO-M group, the RORγt declined by 60%, in the CEO-H group by 67%, and in the mesalazine group by 87%. Compared with the model group, there was an apparent decrease in IL-17A in the CEO treatment groups, and the decrease trend was correlated with the CEO concentration. In the CEO-L, CEO-M and CEO-H groups, the decline was 69%, 86%, and 92%.

With the help of the network pharmacology platform, the relevant pathways and targets of CEO inhibiting *C. albicans* were predicted and verified by relevant experiments. It is concluded that CEO can activate the tyrosine/serine phosphorylation of STAT3 via the cytokine receptor pathway, thereby promoting the synthesis of RORγt. This process helps to balance Th17 and Treg cells, ultimately inhibiting inflammation through IL-6-mediated JAK-STAT signaling. Consequently, CEO plays a key protective role against *C. albicans* infection and in alleviating colitis.

### 3.7. Statistical Analysis

SPSS 26.0 software was used to analyze the variance homogeneity of the experimental results. One-way ANOVA and two-way ANOVA were applied. *p* < 0.05 was considered statistically significant.

## 4. Discussion

*C. albicans* is a polymorphic yeast capable of interacting with the host’s gut microbiota, functioning as both a symbiont and a pathogen, while also serving as a major colonizer within the gastrointestinal tract [37]. CEO demonstrated inhibitory activity against *C. albicans* and alleviated UC in mice colonized by the fungus. In vitro, CEO suppressed *C. albicans* hyphal formation and biofilm development. In vivo, CEO modulated intestinal flora, decreased inflammatory cytokines (IL-6, IL-8, IL-17A), and repaired mucosal damage.

The experimental results show that, as the dose of CEO increased, the DSS-induced UC mice colonized by *C. albicans* showed progressive overall improvement, lethargy and disheveled fur were replaced by increased activity and improved coat condition; the fecal fungal load declined step-wise, with almost no culturable *C. albicans* recovered in the high-dose group; the colonic tissue shifted from diffuse ulceration and dense leukocyte infiltration to smooth mucosa with restored epithelial continuity; and the concomitant decreases in local IL-6, IL-8, and IL-17A levels indicated a steady alleviation of mucosal inflammation [38]. Our findings on the gut microbiota are consistent with the well-documented dysbiosis characteristic of UC. It is widely reported that the normal healthy gut microbiota is dominated by Firmicutes and Bacteroidetes, with a high relative abundance of beneficial genera such as Lactobacillus. In contrast, UC is consistently associated with a marked disruption of this ecosystem. As observed in our model group and supported by other studies, DSS-induced colitis leads to a significant decrease in microbial diversity and a shift in composition, typically characterized by a reduction in Firmicutes and an expansion of Proteobacteria and other pro-inflammatory taxa. Our data align with this pattern: the DSS+C.A model induced marked dysbiosis characterized by reduced overall species richness (as shown by the Venn diagram), a decreased proportion of Firmicutes, and a loss of beneficial genera like Lactobacillus and *Odoribacter*. Concurrently, we observed an increase in the abundance of Bacteroides and other taxa associated with inflammation. After CEO administration, this dysbiotic state was significantly reversed. The microbial diversity gradually recovered, and the overall community structure in the CEO-treated groups, particularly the medium-dose group, moved closer to that of healthy controls. CEO treatment effectively reshaped the intestinal microbiota, markedly increasing the proportion of Firmicutes and reducing the abundance of pro-inflammatory genera. This restorative effect on the gut microbiome is a crucial mechanism through which CEO may confer its therapeutic benefits, as a healthy microbiota is essential for maintaining intestinal barrier integrity and immune homeostasis [39]. Collectively, the disease-model results indicate that CEO exhibits a dose-dependent protective effect in DSS-induced *Candida*-colonized UC mice, alleviating symptoms and exerting therapeutic efficacy.

CEO holds promise for alleviating UC linked to *C. albicans*. During infection, fungal hyphae breach the epithelium and trigger apoptosis, disrupting the barrier integrity and permitting luminal antigen leakage. Concurrently, fungal overgrowth fuels an IL-17A-dominated Th17 response that recruits neutrophils and destroys crypts [40]. CEO dampens IL-17A transcription and downregulates its master transcription factor RORγt, tilting the immune balance toward regulatory T-cell homeostasis. Mechanistically, CEO blocks the IL-6-mediated JAK2–STAT3 signaling axis, uncoupling the fungal sensing from downstream pro-inflammatory gene transcription. These effects establish CEO as a multi-target agent that suppresses fungal virulence, restores barrier function, and reprograms pathogenic immunity within a single therapeutic framework [41].

Although this study confirmed the therapeutic effects of CEO on *C. albicans*-colonized UC and identified possible signaling pathways, several limitations remain to be addressed in future work. As a volatile oil from cinnamon, CEO has a variety of components, although its main component, cinnamaldehyde, has been clarified, how do the other components in CEO play their respective activities and functions? How do these components interact with each other and work together to fight *C. albicans* and regulate gut flora? The action target of CEO and its main components are what we will focus on in the future [42].

## 5. Conclusions

This study completely confirmed that CEO can effectively eliminate *C. albicans* biofilm and inhibit hyphal formation in vitro, and in vivo, it blocks the JAK2-STAT3-IL-17A axis to alleviate UC induced by *C. albicans*. In conclusion, CEO effectively inhibits *C. albicans* biofilm formation in vitro and alleviates *C. albicans*-associated ulcerative colitis in vivo. As a natural, safe, and edible essential oil, CEO has the potential for further development in food and medicinal applications.

## Figures and Tables

**Table 1:** Primer sequences for JAK/STAT signaling pathway–related genes.

Primer Name	Sequences (5′ to 3′)	Number of Bases
JAK2-F	CGCACCATTACCTCTGC	17
JAK2-R	ACCCGCCTTCTTTAGTTTG	19
STAT3-F	GACCCAGGAACAAGGTGA	18
STAT3-R	GCCAAGGAGAGGGAAAGT	18
RORγt-F	CTGTCCTGGGCTACCCTACT	20
RORγt-R	GAAGAAGCCCTTGCACCCC	19
IL-17A-F	TTCACTTTCAGGGTCGAGA	19
IL-17A-R	GGGGTTTCTTAGGGGTCA	18
IL-6-F	ACGAAGTGACGCTCTTGGTA	20
IL-6-R	CGGCTCAGGTATCTCAGTCTT	21
ACTB-F	GGACTGGAGAGGTGGTAGAAC	21
ACTB-R	GTGGAGACAACAGCATCTTCAG	22

## Data Availability

The original contributions presented in the study are included in the article, further inquiries can be directed to the corresponding author.
